# High estrogen induces trans-differentiation of vascular smooth muscle cells to a macrophage-like phenotype resulting in aortic inflammation via inhibiting VHL/HIF1a/KLF4 axis

**DOI:** 10.18632/aging.205904

**Published:** 2024-06-05

**Authors:** Ruijing Zhang, Heng Wang, Xing Cheng, Keyi Fan, Tingting Gao, Xiaotong Qi, Siqi Gao, Guoping Zheng, Honglin Dong

**Affiliations:** 1 Department of Nephrology, The Second Hospital of Shanxi Medical University, Taiyuan, Shanxi, China; 2 Department of Vascular Surgery, The Second Hospital of Shanxi Medical University, Taiyuan, Shanxi, China; 3 Centre for Transplant and Renal Research, Westmead Institute for Medical Research, The University of Sydney, Sydney, NSW, Australia

**Keywords:** estrogen, vascular smooth muscle cells, phenotypic switching, vascular inflammation, Takayasu arteritis

## Abstract

Estrogen is thought to have a role in slowing down aging and protecting cardiovascular and cognitive function. However, high doses of estrogen are still positively associated with autoimmune diseases and tumors with systemic inflammation. First, we administered exogenous estrogen to female mice for three consecutive months and found that the aorta of mice on estrogen develops inflammatory manifestations similar to Takayasu arteritis (TAK). Then, *in vitro* estrogen intervention was performed on mouse aortic vascular smooth muscle cells (MOVAS cells). Stimulated by high concentrations of estradiol, MOVAS cells showed decreased expression of contractile phenotypic markers and increased expression of macrophage-like phenotypic markers. This shift was blocked by tamoxifen and Krüppel-like factor 4 (KLF4) inhibitors and enhanced by Von Hippel-Lindau (VHL)/hypoxia-inducible factor-1α (HIF-1α) interaction inhibitors. It suggests that estrogen-targeted regulation of the VHL/HIF-1α/KLF4 axis induces phenotypic transformation of vascular smooth muscle cells (VSMC). In addition, estrogen-regulated phenotypic conversion of VSMC to macrophages is a key mechanism of estrogen-induced vascular inflammation, which justifies the risk of clinical use of estrogen replacement therapy.

## INTRODUCTION

Estrogens are a class of sex hormones produced primarily by the ovaries and placenta, including estradiol, estrone and estriol. Estrogen plays an important role in slowing down aging. Estrogen has the ability to maintain bone mineral density, maintain the elasticity of blood vessels, preserve skin elasticity and moisture content, and protect cognitive functions of the brain [1–4]. For this reason, estrogen replacement therapy (ERT) is often used to improve various symptoms experienced by postmenopausal women [5]. However, this therapy is not without side effects. Excess estrogen causes overexpression of estrogen receptors (ERα and ERβ), which damages tissues and leads to autoimmune diseases and tumors [6]. Increased cases of breast and ovarian cancer, systemic lupus erythematosus, and multiple sclerosis suggest that the dangers of too much estrogen are worsening [7–9]. This study found that estrogen will induce inflammatory lesions in the aorta of female mice, similar to Takayasu arteritis (TAK).

Vascular smooth muscle cells (VSMCs) are differentiated cells located in the middle layer of arteries that possess contractile function and express marker proteins (e.g., ACTA2, MYH11, and CNN1) [10]. Despite a high degree of differentiation, VSMCs maintain a high degree of plasticity. Under harmful microenvironmental stimuli, VSMCs are activated from a contractile phenotype to a dedifferentiated mesenchymal cell state, in which they further differentiate into fibroblasts, osteoblasts, adipocytes, macrophages, and other characteristic cells [11, 12]. In addition, the plasticity of VSMCs is also closely related to the proinflammatory molecular environment, and this phenotypic transformation ability also induces secondary inflammatory responses [13, 14]. In vascular diseases, such as atherosclerosis [15], vascular calcification [16, 17], diabetes [18], and aortic aneurysms [19], VSMCs exhibit a hypodifferentiated state and dedifferentiate to a macrophage-like phenotype. Macrophage-like VSMCs obtain partial properties of immune cells, incorporating chemotaxis, phagocytosis, and proinflammatory factor release, consequently leading to a chronic inflammatory state in the vessel wall [14, 20]. Thus, increased plasticity of VSMCs is associated with the progression of vascular disease [21].

As research has progressed, emerging factors have been considered to influence the phenotypic transformation of VSMCs. Stimuli that trigger VSMC phenotypic regulation include injury, mechanical force, intracellular microenvironment, extracellular microenvironment, abnormal substance accumulation, and intercellular interactions [22–25]. Given that the prevalence of cardiovascular disease is much lower in premenopausal women than in men or postmenopausal women, estrogen is often considered beneficial to blood vessels [26]. Estrogen also has a role in vasodilating blood vessels, protecting vascular endothelial cells, and repairing damaged blood vessels [27, 28]. Estradiol (E2) is the most predominant and biologically active of estrogens. However, the effects of estrogen on blood vessels are contradictory and controversial [29, 30]. In postmenopausal women, Susana et al. found that, although short-term estrogen intervention exerts an anti-inflammatory effect, long-term use shifts to a proinflammatory state, whereas raloxifene treatment has no proinflammatory effect [31]. In rats with spinal cord injury, Samantaray et al. found that low doses of estrogen reduce inflammation, attenuate cell death, and promote angiogenesis [32]. In addition, estrogen or estrogen receptor (ER) signaling is highly expressed in breast cancer [33], endometriosis [34], and allergic respiratory inflammation [35], and it is positively correlated with systemic inflammation. Zhang et al. exposed rat VSMCs to gradient concentrations of E2 (10^−9^ to 10^−5^ M) and found a concentration-dependent promotion of VSMCs growth in the range of physiological levels, while they reported that high concentrations of E2 have a growth inhibitory effect [36].

Krüppel-like factor 4 (KLF4) is a member of the zinc-finger transcription factor family required for mammalian embryonic development and various diseases. KLF4 acts as a key initiator responsible for the dedifferentiation of VSMCs from a contractile phenotype to mesenchymal-like phenotype [37, 38]. Von Hippel-Lindau (VHL) is a ubiquitin protein ligase that regulates the stability of KLF4 protein. Binding of estrogen to ERα results in ubiquitin-dependent downregulation of VHL, which in turn leads to accumulation of KLF4 [39, 40]. Furthermore, if a binding element for hypoxia-inducible factor-1 alpha (HIF-1α) is found in the upstream regulatory region of the KLF4 locus, then VHL also regulates KLF4 by degrading HIF-1α [41, 42].

Therefore, the aim of the present study was to investigate the effect of estrogen on the phenotypic transformation of VSMCs and its role and potential mechanisms in inflammatory changes in large arteries.

## RESULTS

### High estrogen induces Takayasu arteritis-like symptoms in female mice

**Figure 1:**
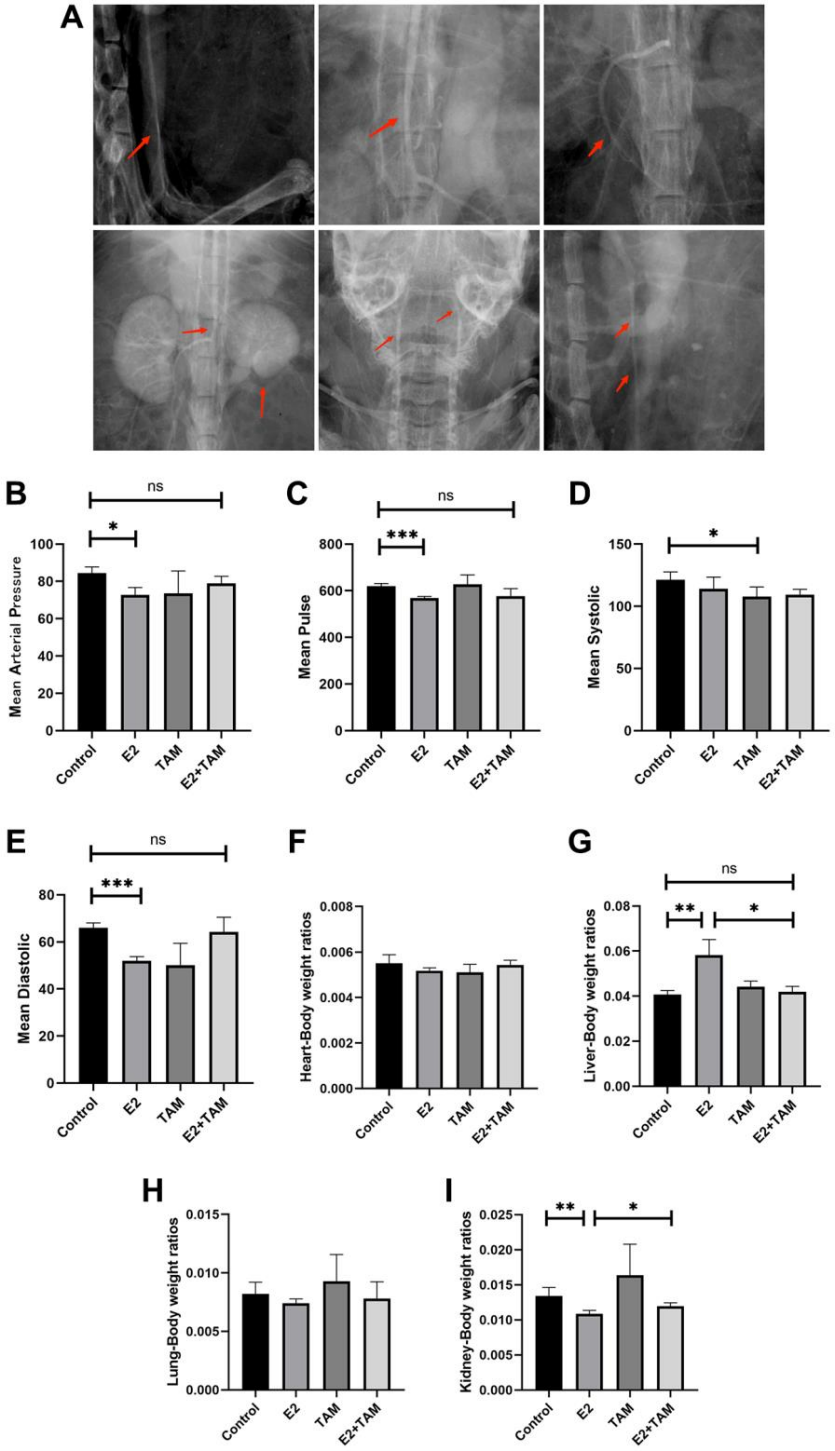
**Estrogen-induced inflammatory changes in the aortas of mice.** (**A**) After three months of continuous intraperitoneal injection of E2 in mice, x-ray angiography was performed to observe the condition of the aorta, branches and organs. (**B**–**E**) Blood pressure and pulse rate in the tail artery of mice were monitored using a noninvasive sphygmomanometer (*n* = 6). (**F**–**I**) After sacrificing the mice, the hearts, livers, lungs, and kidneys were collected, and the organ/body wet-weight ratios were calculated (*n* = 6). ^*^P < 0.05, ^**^P < 0.01, ^***^P < 0.001, indicating statistically significant data between groups; Abbreviation: ns indicates no statistical significance.

Three months after exogenous intraperitoneal administration of E2 to female mice, we performed X-ray angiography on the mice. X-ray angiography confirmed significant stenotic segments in the suprarenal and subrenal abdominal aorta as well as bead-like changes in the carotid artery, changes in the superior mesenteric artery (including stenosis of secondary arteries), and atrophy of the kidney in the E2 group (Figure 1A). Blood pressure testing of mice at the end of model establishment revealed that the mean arterial pressure in the caudal artery of mice in the E2 group was attenuated, mainly due to a decrease in systolic blood pressure, and the pulse rate in the caudal artery was also significantly attenuated (Figure 1B–1E).

Arteritis not only affects localized organs, but may also affect the overall metabolic and nutritional status of the animal. This can lead to weight loss, which is a sign of systemic inflammation or systemic stress. As a result of these changes, changes in organ/body weight ratios may indicate the impact of arteritis on overall health. Measurement and calculation of the organ/body weight wet weight ratios of model mice demonstrated that E2 did not significantly change the mass of the heart and lungs. In addition, E2 significantly increased the liver/body weight wet weight ratio but decreased the kidney/body weight wet weight ratio, which may have been related to the narrowing of the renal artery triggered by E2, resulting in atrophy due to insufficient blood supply to the kidney (Figure 1F–1I).

**Figure 2:**
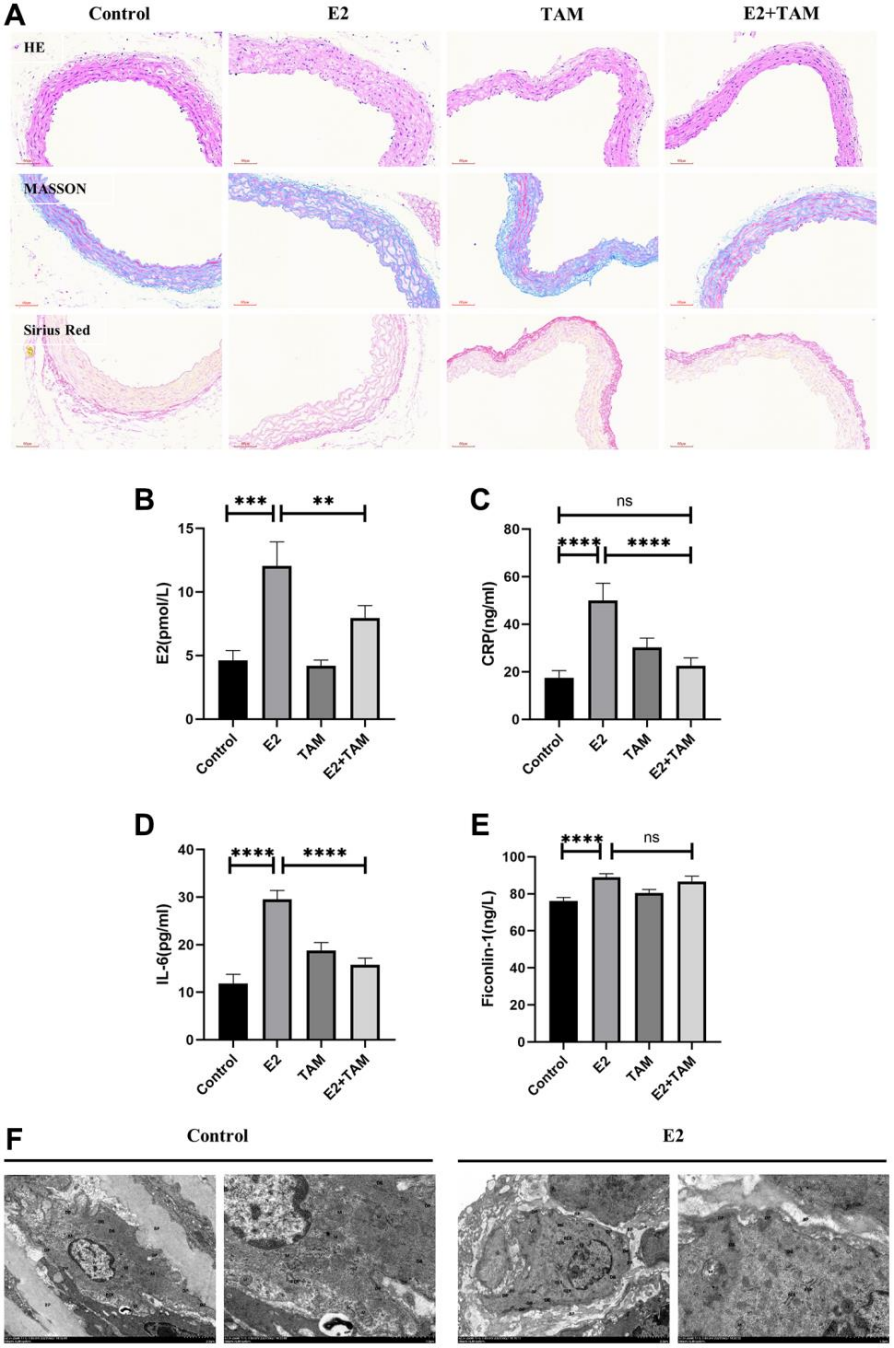
**Detection of pathological changes in the aorta and serum inflammatory markers in mice.** (**A**) The abdominal aorta was examined by pathology using HE, Masson’s trichome, and Picro-Sirius Red staining. (**B**–**E**) Peripheral blood of mice was taken and serum was centrifuged for analysis. E2, CRP, IL-6 and Ficonlin-1 levels were measured by ELISA. (**F**) The abdominal aorta of the model group was observed by TEM, which indicated slight swelling of organelles. The nucleus (N) was irregularly shaped and shrunken. Mitochondria (M) were abundant. The RER showed no obvious dilation and a large number of ribosomes attached to the surface. DPs and DBs were abundant and evenly distributed. Magnification 2500× and 7000×. ^**^P < 0.01, ^***^P < 0.001, ^****^P < 0.0001, indicating statistically significant data between groups; Abbreviation: ns indicates no statistical significance.

Pathological staining of mouse abdominal aorta with HE, Masson’s trichrome, and Picro-Sirius Red revealed thickened arterial wall, disorganized smooth muscle layer structure, VSMCs proliferation, loosely arranged collagen fibers, aggregation of proliferating VSMCs into clusters and bundles, and visible hyaline degeneration of cytoplasm in the E2 group (Figure 2A). The detection of mouse serum by ELISA confirmed that serum estrogen, CRP, and IL-6 levels were significantly elevated in the E2 group (Figure 2B–2D). In addition, Ficonlin-1, a potential specific marker for TAK [43, 44], was also highly expressed in the E2 group (Figure 2E).

According to the 2022 American College of Rheumatology/EULAR Classification Criteria for TAK [45]. In summary, C57 female mice intravenously injected with E2 for 3 months showed the following manifestations: (1) abnormal blood pressure and pulse rate in the caudal artery, including decreased blood pressure and reduced pulse rate; (2) inflammatory response as indicated by increased serum levels of CRP and IL-6; (3) elevated expression of Ficonlin-1, a potential serum marker of TAK.; (4) pathological manifestations, including thickening of the wall of the abdominal aorta and hyperplastic degeneration of the smooth muscle layer; (5) organ changes, including atrophy of the kidney; and 6) characteristic imaging manifestations as demonstrated by X-ray angiography, which revealed stenosis in the secondary and higher arterial branches of the abdominal aorta, carotid artery, and superior mesenteric artery. These pathologic manifestations are very similar to TAK.

### Estrogen induces conversion of VSMCs to a macrophage-like phenotype in mice

TEM analysis of the abdominal aorta of E2 group mice (Figure 2F) showed that VSMCs exhibited a synthetic cell state with abundant mitochondria (M). The rough endoplasmic reticulum (RER) did not show significant expansion, and a large number of ribosomal attachments were visible on the surface. Moreover, dense plaques (DPs) and dense bodies (DBs) were abundant, and VSMCs organelles were mildly swollen. In addition, the cytoplasm was reduced, and the nucleus was shrunk. The TEM results demonstrated that VSMCs showed an active synthetic state with increased cellular protein synthesis and secretion, which differed from the stable contractile phenotype (Figure 2F).

**Figure 3:**
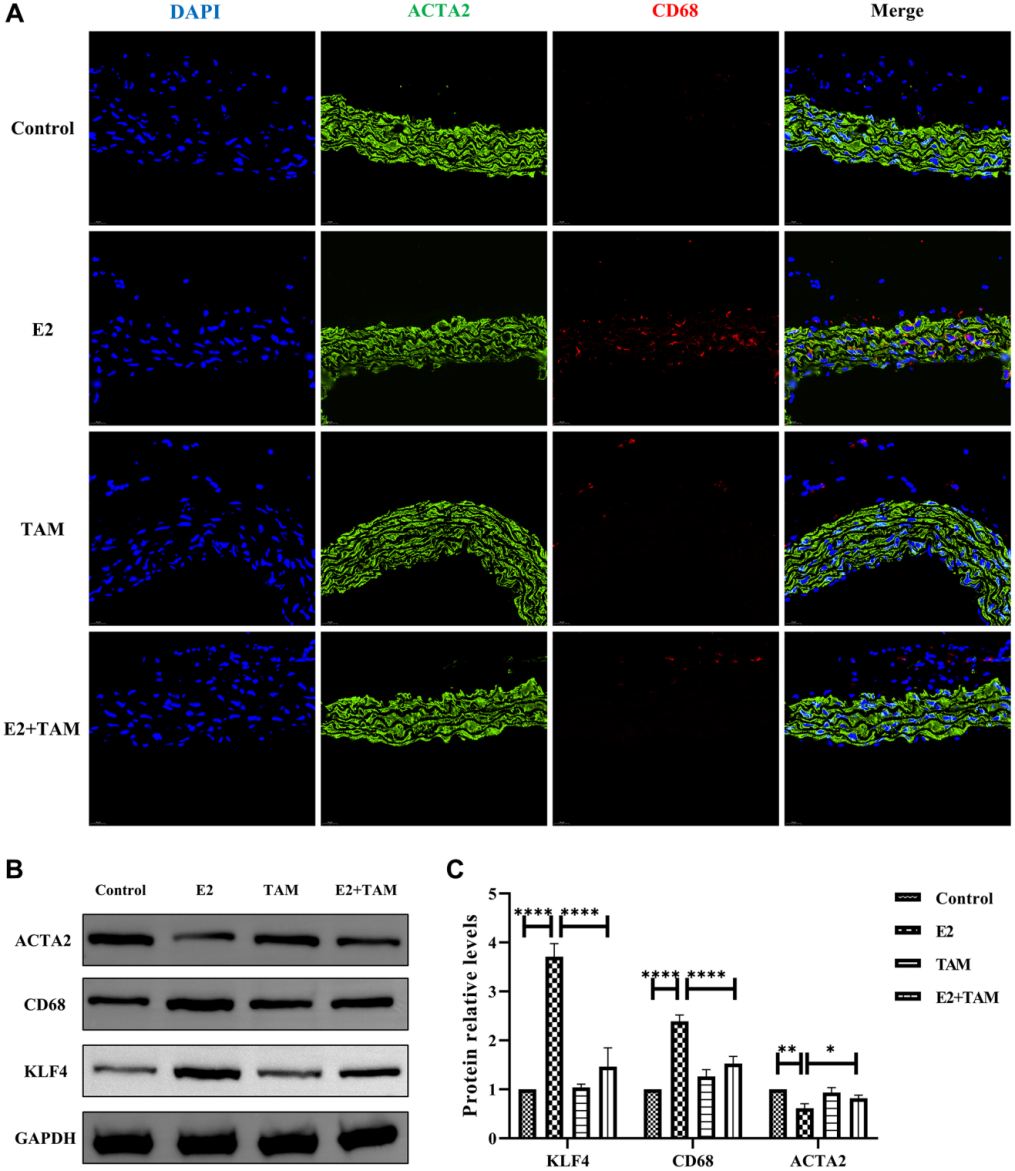
**E2 induces transformation of VSMCs into a macrophage-like phenotype in mice and TAM has protective effect on blood vessels.** (**A**) The paraffin sections of abdominal aorta were stained with ACTA2 (green) and CD68 (red). Magnification 630×. (**B**, **C**) The whole aortic tissue was lysed, and western blot analysis was performed to analyze the protein levels of KLF4, CD68 and ACTA2 (*n* = 4). ^*^P < 0.05, ^**^P < 0.01, ^****^P < 0.0001, indicating statistically significant data between groups.

In the E2 group, double staining of the abdominal aorta with ACTA2 and CD68 revealed expression of the CD68 macrophage marker on VSMCs, which was not found in the normal group (Figure 3A). Meanwhile, aortic F4/80 fluorescence intensity was elevated in the E2 group (Supplementary Figure 1A). Western blot analysis of whole mouse aortas revealed increased expression of KLF4, a key protein for VSMC phenotypic transformation, in the E2 group (Figure 3B, 3C and Supplementary Figure 2). In addition, the expression of contractile phenotype-related proteins, ACTA2 was reduced, while the expression of macrophage marker proteins, CD68 was increased (Figure 3B, 3C).

This suggests that administration of exogenous estrogen to female mice induces the conversion of vascular smooth muscle cells to a macrophage-like phenotype.

### Inhibition of estrogen with TAM improves the manifestation of aortic inflammation in mice *in vivo*

TAM antagonizes the effect of E2 by binding to ESR1. In the mouse model of polyarteritis, TAM ameliorated the E2-induced attenuation of mean arterial pressure and slowing of pulse rate in the tail artery of mice (Figure 1B–1E). Regarding the organ/body weight wet weight ratio, TAM treatment alone, which had no significant effect on organ weight in mice, attenuated the E2-induced weight gain of the liver and did not show E2-induced kidney atrophy (Figure 1F–1I). In addition, TAM reduced the high estrogen, CRP, and IL-6 levels induced by E2, and it also attenuated the inflammatory response. However, there was no significant change in Ficonlin-1 levels in the E2+TAM group compared to the E2 group (Figure 2B–2E).

Pathological staining of the abdominal aorta of mice with HE, Masson’s trichome, and Picro-Sirius Red was performed, which demonstrated that the abdominal aorta of mice in the E2+TAM group was similar to the normal group without the pathological manifestations associated with the E2 group (Figure 2A). Compared to the E2 group, the protein expression of ACTA2 (contractile phenotype) was increased in the E2+TAM group, while the protein expression of CD68 (macrophage marker) was decreased in the E2+TAM group (Figure 3B, 3C). In addition, the expression of KLF4 was lower in the E2+TAM group compared to the E2 group (Figure 3B, 3C). And in immunofluorescence staining, TAM decreased the expression of CD68 (Figure 3A and Supplementary Figure 1B).

Therefore, these findings indicated that TAM ameliorates the pathological manifestations associated with TAK in mice by inhibiting the conversion of VSMCs to a macrophage-like phenotype.

### Estrogen-induced conversion of MOVAS cells to a macrophage-like phenotype

**Figure 4:**
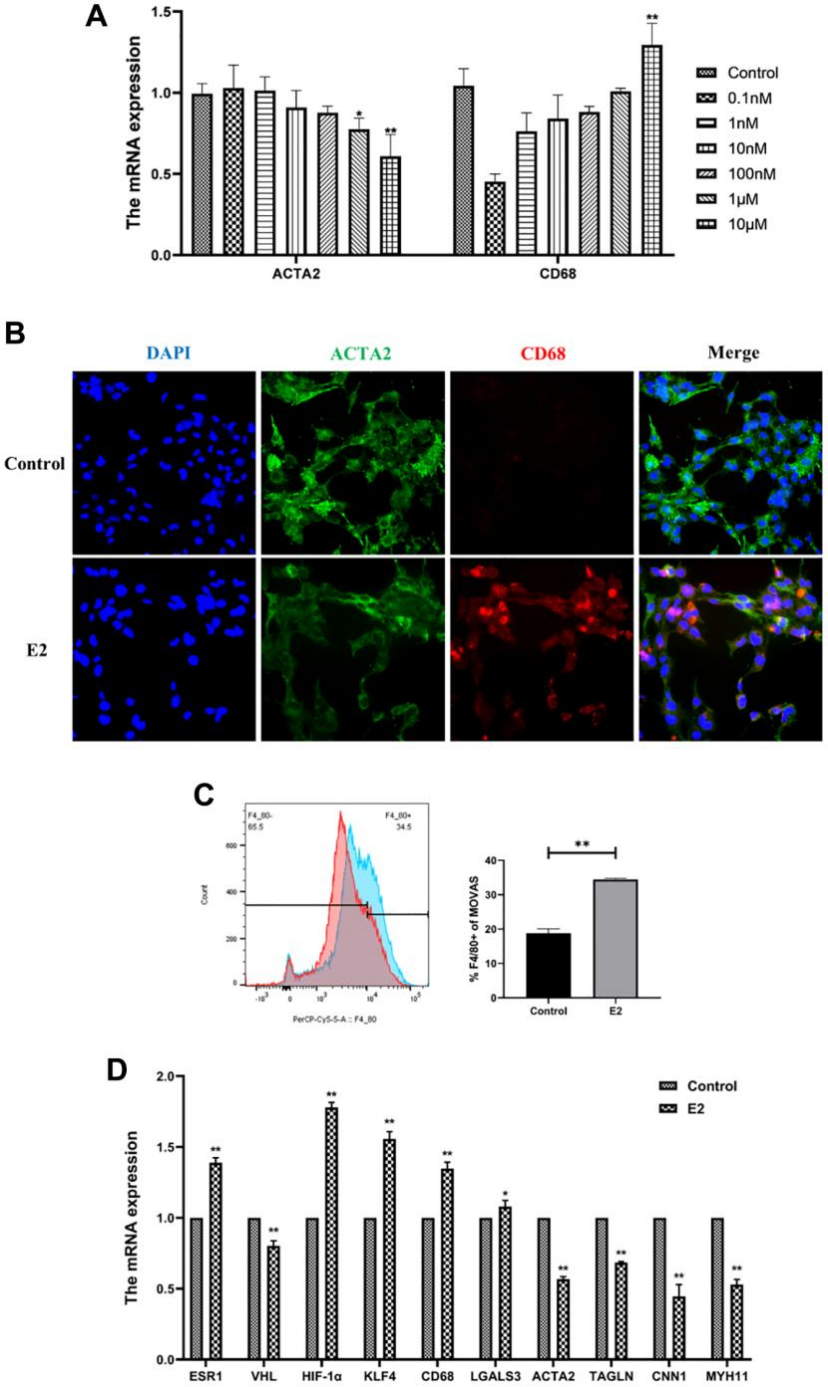
**E2 induces transformation of MOVAS cells into a macrophage-like phenotype.** (**A**) MOVAS cells were treated with E2 (0.1 nM, 1 nM, 10 nM, 100 nM, 1 μM, and 10 μM) for 4 days. The contractile phenotype marker, ACTA2, and the macrophage marker, CD68, were detected by qRT-PCR. (**B**–**D**) MOVAS cells were treated with 10 μM E2 for 4 days (*n* = 4). (**B**) ACTA2 and CD68 levels in MOVAS cells were detected by immunofluorescence analysis. (**C**) F4/80 antibody expression on the surface of MOVAS cells was detected by FCM analysis (*n* = 3). (**D**) Contraction phenotype-related markers (ACTA2, TAGLN, CNN1, and MYH11) and macrophage-like phenotype-related markers (CD68 and LGALS3) in MOVAS cells were evaluated by qRT-PCR (*n* = 4). ^*^P < 0.05, ^**^P < 0.01, indicating statistically significant data between groups.

Estrogen affected the phenotype of VSMCs in a concentration- and time-dependent manner. The present study confirmed that low concentrations of estrogen maintained the contractile phenotype of VSMCs, while higher concentrations of estrogen induced significant changes in the phenotype of MOVAS cells. After treatment of MOVAS cells with 0.1 nM, 1 nM, 10 nM, 100 nM, 1 μM, and 10 μM E2 for 4 days, and the mRNA expression of ACTA2 and CD68 was detected. The results indicated that 10 μM E2 induced the phenotypic transformation of MOVAS cells (Figure 4A).

Treatment of MOVAS cells with 10 μM E2 for 4 days significantly upregulated CD68 expression in MOVAS cells according to immunofluorescence analysis (Figure 4B). FCM analysis demonstrated that estrogen induced a significant increase in F4/80+ MOVAS cells (Figure 4C). In addition, qRT-PCR indicated that the mRNA expression levels of smooth muscle contraction-related genes, namely, ACTA2, TAGLN, MYH11, and CNN1, were decreased, while the mRNA expression levels of macrophage-related genes, namely, CD68 and LGALS3, were increased (Figure 4D).

This suggests that E2 can induce MOVAS cells to express macrophage markers in an *in vitro* cellular assay.

### High estrogen induces phenotypic trans-differentiation of vascular smooth muscle cells by inhibiting VHL/HIF1a/KLF4 axis

**Figure 5:**
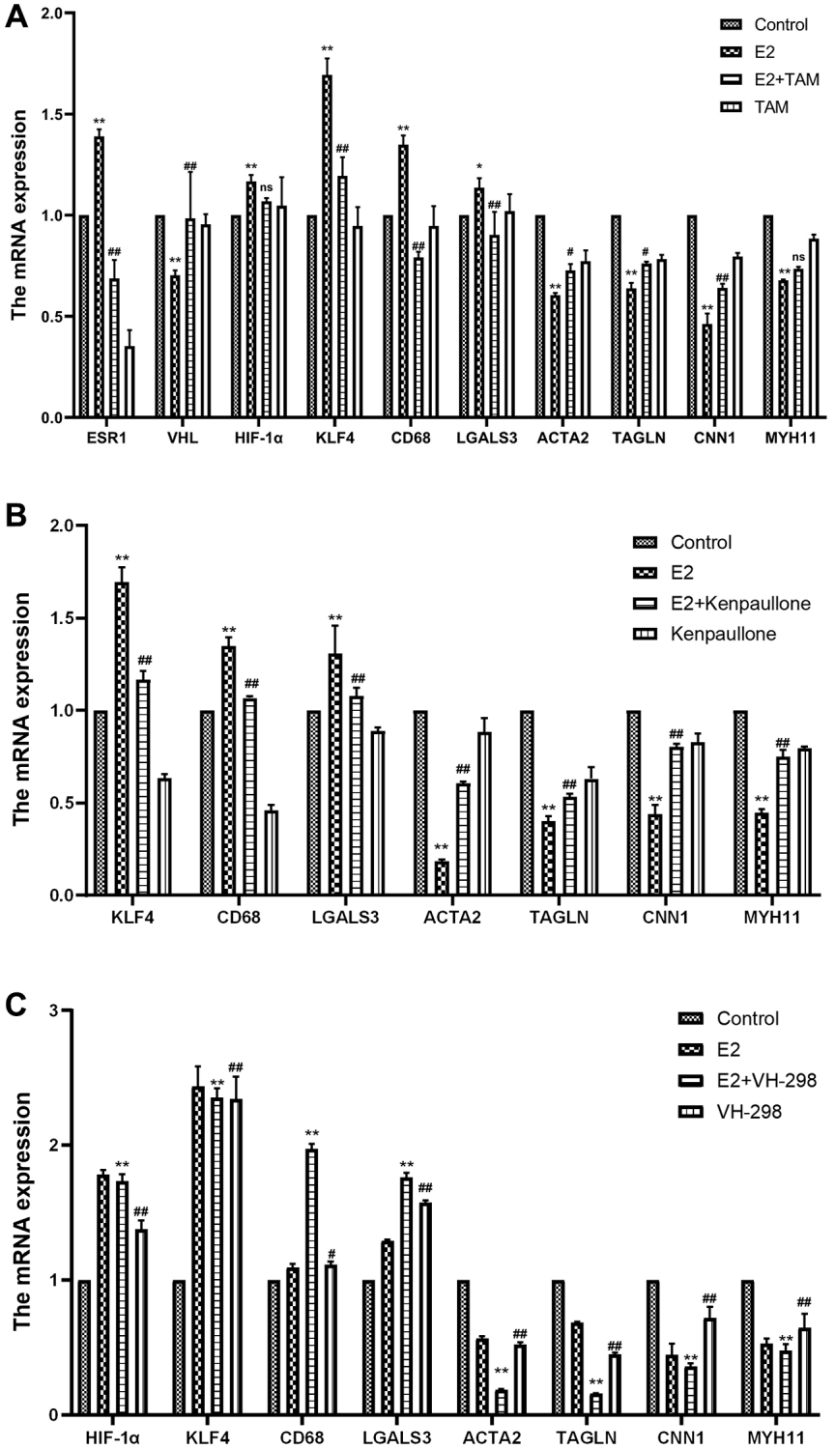
**Effect of high-dose estrogen on the expression of MOVAS cell markers.** (**A**) MOVAS cells were treated with 10 μM E2 with or without TAM. After 4 days, the contraction phenotypic markers and macrophage-like phenotypic markers of MOVAS cells were detected by qRT-PCR as well as the mRNA expression of related cellular pathways (*n* = 4). (**B**) MOVAS cells were treated with 10 μM E2 with or without kenpaullone. After 4 days, the mRNA expression levels of related genes were detected by qRT-PCR (*n* = 4). (**C**) MOVAS cells were treated with 10 μM E2 with or without VH-298 for 4 days, and the mRNA expression levels of related genes were detected by qRT-PCR (*n* = 4). ^*^VS control group, ^*^P < 0.05, ^**^P < 0.01, indicating statistically significant data between groups; ^#^VS. E2 group, ^#^*P* < 0.05, ^##^*P* < 0.01, indicating statistically significant data between groups; ns indicates no statistical significance.

**Figure 6:**
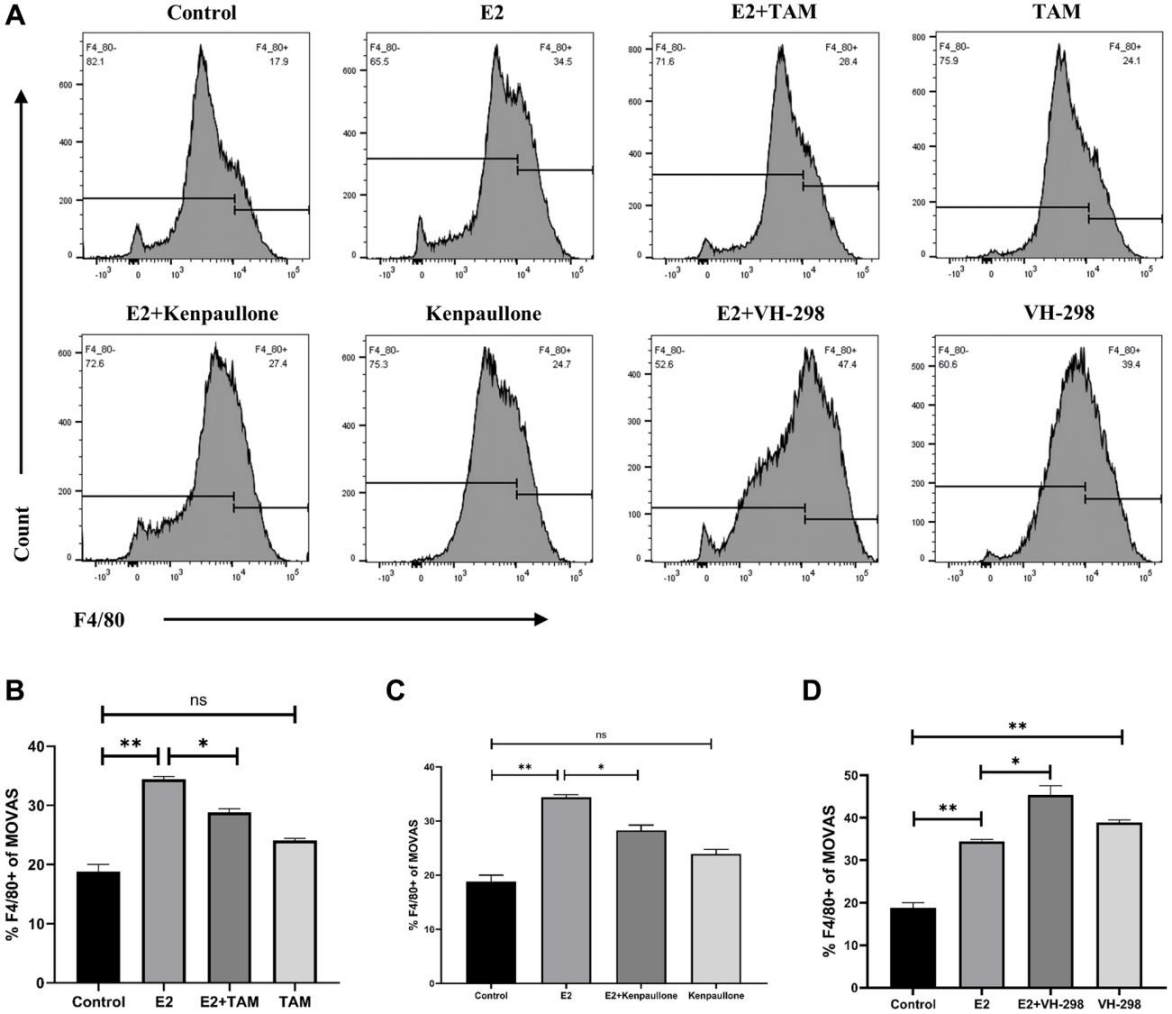
**E2 induces phenotypic transformation by regulating the VHL/HIF-1α/KLF4 axis.** (**A**–**D**) MOVAS cells were treated with 10 μM E2 for 4 days with or without the following inhibitors: Tamoxifen (1 μM), kenpaullone (3 μM), and VH-298 (10 μM). The expression of the F4/80 macrophage marker on MOVAS cells was detected by FCM analysis (*n* = 3). ^*^P < 0.05, ^**^P < 0.01, indicating statistically significant data between groups; ns indicates no statistical significance.

ERα is a novel proteasomal degradation target of the VHL E3 ligase. In the present study, high levels of estrogen increased the expression levels of ERα and increased the degradation rate of VHL. Tamoxifen (TAM), an estradiol competitive antagonist, bound to ESR1 and inhibited the degradation of VHL (Figure 5A). Thus, these results indicated that TAM inhibits the conversion of MOVAS cells to a macrophage-like phenotype and decreases the F4/80+ cell percentage (Figure 6A, 6B). In addition, TAM decreases HIF-1α expression in mouse aortic sections (Supplementary Figure 3).

Kenpaullone is a small molecule inhibitor of KLF4. Treatment with kenpaullone significantly reduced the mRNA expression of E2-induced macrophage-related genes (Figure 5B) and significantly reduced the number of F4/80+ cells in MOVAS cells as detected by FCM analysis (Figure 6A, 6C).

VH-298 is a highly efficient inhibitor of the VHL/HIF-α interaction, and it induces the production of HIF-1α by blocking the VHL-triggered hypoxic response, which in turn causes the accumulation of KLF4 and initiates the phenotypic transformation of MOVAS cells. Treatment of MOVAS cells with VH-298 alone decreased the expression of genes associated with the contractile phenotype but increased the expression of genes associated with the macrophage-like phenotype (Figure 5C). This promotion of phenotypic switching was enhanced by cotreatment with E2. FCM analysis demonstrated that E2+VH-298 treatment increased the percentage of F4/80+ MOVAS cells (Figure 6A, 6D).

These results suggested that E2 induces the conversion of MOVAS cells to a macrophage-like phenotype by regulating the VHL/HIF-1α/KLF4 axis.

## DISCUSSION

**Figure 7:**
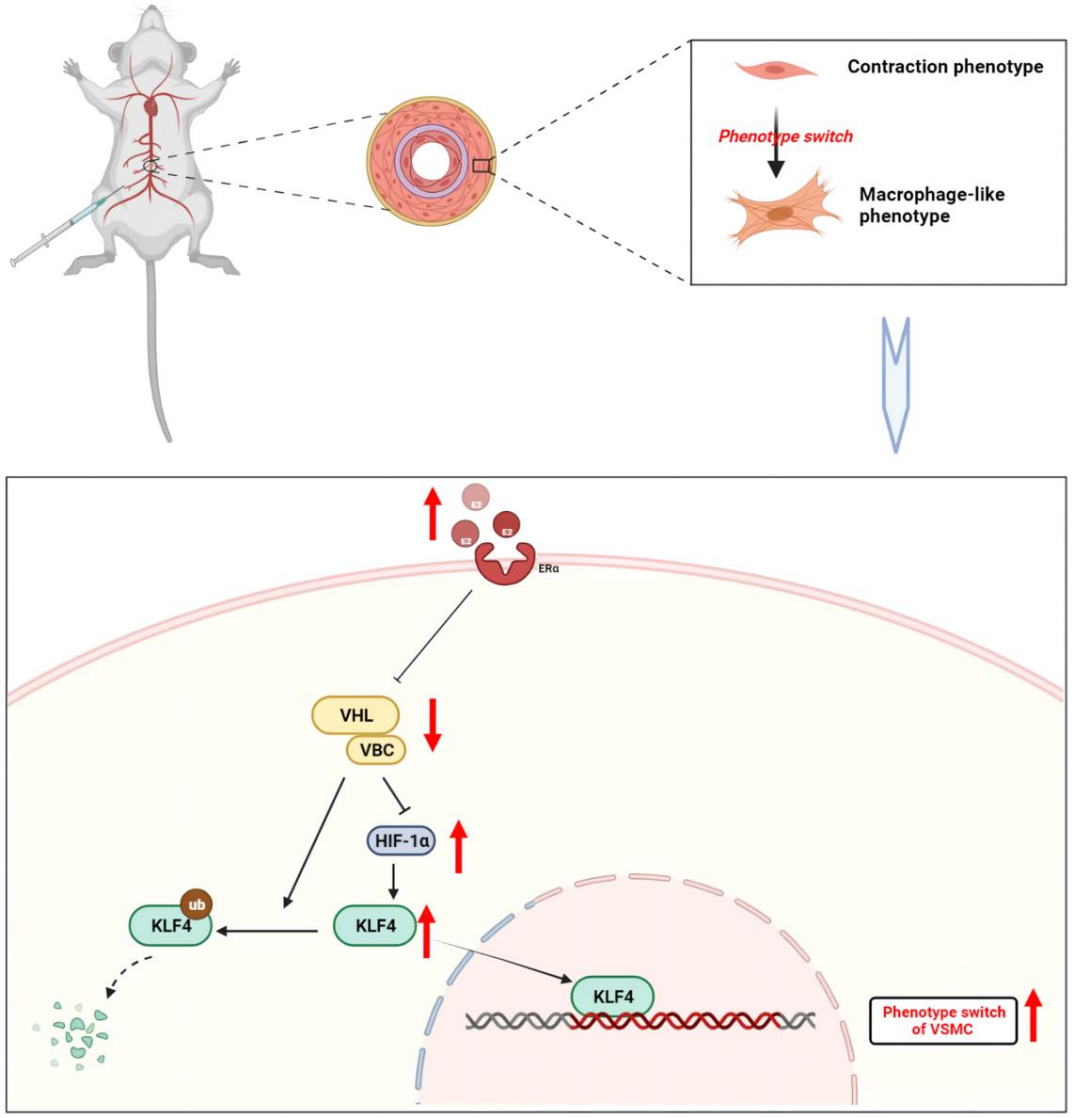
**Mechanism of estrogen-induced conversion of VSMCs to a macrophage-like phenotype leading to inflammation in mouse aorta.** Upon binding to its receptor, estrogen inhibits the binding of VHL/VBC. VHL induces both ubiquitination degradation of KLF4 and acts to hydrolyze HIF-1α. When VHL expression is reduced, KLF4 expression rises, which in turn enters the nucleus and participates in the phenotype of vascular smooth muscle cells. Created with BioRender.

KLF4 plays a key role in VSMC phenotypic transformation. For the first time, the present study demonstrated that estrogen activated KLF4 by regulating VHL/HIF-1α, which in turn promoted phenotypic transformation of MOVAS cells. Estrogen treatment initiated the transformation of VSMCs to a macrophage-like phenotype by downregulating VHL and reducing the degradation of HIF-1α, thereby inducing the accumulation of the KLF4 transcription factor (Figure 7).

Large vessel vasculitis mainly includes TAK and giant cell arteritis (GCA), in which the histological lesions show an inflammatory infiltrate mainly located in the tunica media and tunica adventitia of the aorta [46]. VSMCs are major components of the media, and their phenotypic transformation influences the development of vascular inflammatory lesions [47]. TAK is a rare vasculitis that occurs primarily in young women and is characterized by a chronic idiopathic inflammation involving the aorta and its associated branches [48]. Because TAK presents with non-specific symptoms in the acute early stage (headache, fever, and muscle pain), the diagnosis of TAK is often delayed until the onset of substantial arterial injury [49]. TAK initially triggers thickening, fibrosis, and thrombosis of the vessel wall, which may progress to stenosis, occlusion, dilatation, or aneurysm of the affected artery, and it may even be life-threatening [50]. The etiology of TAK is unclear, but there is evidence that pathogenic T cells and macrophages contribute to the chronic inflammatory lesion in the presence of genetic susceptibility, environmental triggers, microorganisms, pathogens, and their antigenic components [51–53]. Given that the proportion of female patients is 5–12 times higher than that of male patients, estrogen is considered an independent risk factor for the pathogenesis of TAK [54]. Most patients with TAK have been diagnosed with hyperestrogenemia. Hyperestrogen exerts a degenerative, atrophic, and profibrotic effect on VSMCs, and the thrombogenic properties of estrogen may also contribute to the development of TAK [55]. In addition, supplementation of exogenous estrogen to female rats induces a disease model similar to human TAK, suggesting that estrogen induces aortic inflammation [56]. Because research on the correlation between estrogen and large vessel inflammation is lacking, the present study investigated the mechanism of estrogen-induced inflammatory changes in large arteries of mice.

By referring to various diagnostic criteria for multiple aortitis, such as the 1990 American College of Rheumatology (ACR) criteria [57], the 2010 European EULAR/PRES/PRINTO criteria [58], and the 2015 Chinese diagnostic criteria for aortitis [59], the present animal model was considered as a mouse model of multiple aortitis. In particular, angiography revealed stenosis of the abdominal aorta, superior mesenteric artery, and carotid artery in mice accompanied by atrophy of the kidneys, supporting successful establishment of the TAK model in mice. For the first time, the present study demonstrated that high doses of estrogen induce VSMCs to convert to a macrophage-like phenotype in this model.

We have previously reported that the transformation of VSMCs to a macrophage-like phenotype occurs in a mouse model of abdominal aortic aneurysm and is associated with chronic inflammation of blood vessels [19, 60]. In contrast, vascular inflammatory diseases are often associated with high estrogen levels and are epidemiologically more prevalent in women of childbearing age [61]. For example, in systemic lupus erythematosus (SLE), estrogen exacerbates the inflammatory response by targeting key immune pathways, including type 1 interferon (IFN) responses, differentiation of CD4+ T helper cells, and survival of self-reactive B cells [62]. Regarding the development of TAK, estrogen plays a pivotal role as high estrogen levels and sustained action of estrogen are involved in the mechanism of TAK injury by inducing the conversion of VSMCs to a macrophage-like phenotype.

KLF4 is a zinc-finger transcription factor that plays a key role in cell proliferation and differentiation in physiological and pathological states, and KLF4 is thought to be a switch for VSMC phenotypic transformation [63]. Transcription of SMC contractile phenotype marker genes is controlled by common cis-acting elements, including multiple CC(A/T)6GG(CArG) elements and a transforming growth factor-β control element (TCE) [63]. KLF4, a transacting factor of TCE, is overexpressed and promotes phenotypic transformation by binding to the TCE in response to the stimulus of induced VSMC phenotypic transformation [64]. In addition, epigenetic regulation is also an important factor determining the differentiation status of VSMCs, especially in the field of histone acetylation and methylation [65]. Interestingly, when a VSMC initiates transformation, the exact type of cell into which it transforms is uncertain and may depend on the stimulus. The present study found that high doses of estrogen induce VSMCs to express macrophage markers, but we did not explore whether this translates into other cellular phenotypes.

During estrogen-induced phenotypic transformation of VSMCs, estrogen leads to VHL degradation by binding to ERα, and the regulation of VHL on KLF4 is not dependent on HIF-1α. VHL directly targets KLF4 for ubiquitination degradation and also acts as a substrate specific splice for the VCB-Cul2 E3 ubiquitin ligase complex. The complex targets HIF-1α for protein hydrolysis, and HIF-1α has two binding elements at the upstream regulatory region motif of KLF4, which can be targeted to induce upregulation of KLF4 [66, 67]. Sivritas et al. treated rat aortic VSMCs with estrogen and found a rapid increase in KLF4 expression and a 50% reduction in cell proliferation after estrogen binding to Erα [68]. These results suggest that estrogen may produce an inhibitory effect on VSMCs proliferation and induce differentiation, which is contrary to previous knowledge.

Although estrogen is generally considered to have a protective effect on blood vessels, adverse effects of estrogen on blood vessels have been reported in recent years. First, estrogen has age-related inflammatory effects on blood vessels. E2 is cardioprotective in young females and postmenopausal women, but vasotoxic effects of E2 have been observed in older females and postmenopausal women [69], which may be related to age-dependent regulation of ERs as older women have reduced expression of ERα and increased expression of ERβ and the novel GPR30 ER [31, 70]. Second, estrogen has differential effects on large vessels and microvessels, which is attributed to different non-genomic mechanisms. In general, estrogen exerts its early anti-inflammatory effects on large vessels probably through inhibition of NF-κB expression, while it exerts its pro-inflammatory effects on microvessels by increasing adhesion molecules (ICAM-1 and VCAM-1) [70]. These results are contradictory to the present study but may be relevant to the pathogenesis of acute and chronic inflammation. Estrogen increases vascular NO production by activating eNOS and iNOS, and the resulting changes in NO concentration determine the effect on inflammation [71]. Thus, estrogen may have an early acute anti-inflammatory effect on the vasculature, which then migrates into a chronic proinflammatory process. Third, there are different ER-mediated signaling pathways. Estrogen has two main receptors, namely, ERα and ERβ. It has been reported that long-term exposure to estrogen upregulates proinflammatory ERα expression in endothelial cells (ECs) (e.g., fetal sheep placental internal arteries) but downregulates anti-inflammatory ERβ expression in human endothelium [72, 73]. In addition, the anti-inflammatory mechanism mediated by the novel GPR30 ER is not fully understood [28]. Fourth, high levels of estrogen have adverse effects on blood vessels. Small doses of estrogen reduce the persistent inflammatory response and promote angiogenesis after chronic spinal cord injury in rats, whereas high doses of estrogen do not have this effect [32]. In addition, natural estrogens have anti-inflammatory properties, while synthetic estrogens have proinflammatory and carcinogenic effects [74]. Barreno et al. first reported inflammatory changes and atherosclerosis in the blood vessels of the testes and epididymis in men taking long-term exogenous estrogens [75]. Therefore, the effects of estrogen levels, as well as the duration of exposure, on the vasculature still need further investigation.

As phytoestrogens have been identified as a natural, plant-based alternative to synthetic sources of estrogen. Over the past 10–15 years, there has been increasing interest in the possibility of estrogen supplementation through diet and supplements in order to improve estrogen levels in postmenopausal women [76, 77]. A growing body of evidence suggests that the consumption of these plant-derived compounds can prevent the onset or attenuate the progression of pathological conditions such as aging, decline in mental acuity, dysmetabolism including atherosclerosis and diabetes, stroke, menopausal symptoms, and osteoporosis [78]. In terms of nutrition, the nutritional effects of estrogen on the uterus and the antioxidant effects of phytoestrogens have also received attention from researchers [79, 80]. It is worth noting that, based on the results of this experiment, there are still potential risks during estrogen application.

ERT, a treatment by supplementing or replacing estrogen in the body, has been used for a long time as a mainstream therapy for disease prevention in postmenopausal women, for the prevention of menopausal osteoporosis, enhancement of immune function, and improvement of symptoms of endocrine disorders in menopausal patients. Estrogen replacement therapy has the following advantages: (1) Relief of menopausal symptoms: ERT can help relieve menopausal symptoms such as insomnia, hot flashes, and vasodilatory instability (i.e., night sweats). Hot flashes may be due to disruption of the thermoregulatory center caused by a decrease in estrogen levels, leading to increased vasodilation and sweating [81]. (2) Improvement of metabolic syndrome: ERT is beneficial for blood pressure, glycemic control, low density lipoprotein, cholesterol, triglycerides and lipoprotein a [82]. (3) Protect bone density: Estrogen has a protective effect on bones, slowing the loss of bone density and strength and reducing the risk of pathologic fracture breaks. (4) Improve cognition: cognitive decline and dementia syndromes such as Alzheimer's disease can be prevented in postmenopausal women through the use of ERT [83]. (5) Reducing the risk of cardiovascular disease: estrogen promotes blood vessel formation and protects the heart from ischemic damage; estrogen prevents endothelial damage, platelet aggregation, and plaque formation, which ultimately reduces the risk of atherosclerosis; estrogen has demonstrated cardiovascular benefits in several research trials, but the mode of administration and the age of initiation are still topics of discussion [84]. In addition, estrogen replacement therapy has the following disadvantages: (1) Increased risk of breast cancer: long-term use of ERT may increase the risk of breast cancer. (2) Increased risk of endometrial cancer: long-term use of ERT may increase the risk of endometrial cancer in women who have not yet had their uterus removed. 3) Cardiovascular Disease Risk: Excessive ERT may increase the risk of stroke and venous thromboembolism, especially in older women with pre-existing cardiovascular disease or high-risk factors [85]. In conclusion, estrogen replacement therapy has advantages in relieving menopausal symptoms and protecting bone density, but its role in cardiovascular disease is unclear.

## CONCLUSION

In summary, the present study demonstrated that estrogen induces MOVAS cells to transform into a macrophage-like phenotype in a dose- and time-dependent manner. In addition, high dose and prolonged estrogen treatment induces inflammatory lesions in large arteries in female mice similar to human TAK. Moreover, estrogen binding to ERα targets VSMC phenotypic transformation through the VHL/HIF-1α/KLF4 axis. These data suggested that estrogen may play a key role in the development of TAK by regulating the phenotype transformation of VSMCs to a macrophage-like phenotype, providing new insights into the mechanisms of phenotypic transformation in vascular disease.

## MATERIALS AND METHODS

### Animal treatment

Female C57B6/J mice (6–8 weeks old and weighing 20 ± 2 g) were purchased from the Shanxi Medical University Experimental Animal Center (Taiyuan, China) and maintained in a specific pathogen-free environment at our facility (SYXK 2021-0001). All animals were fed standard food and had free access to water. All animal experiments were conducted in a humane manner and according to the Institutional Animal Care Instructions. The mice were randomly divided into the following four groups: control, no treatment; E2, 400 μg/kg estrogen; TAM, 10 mg/kg Tamoxifen; E2+TAM, combined treatment of 400 μg/kg estrogen and 10 mg/kg Tamoxifen. Stock solutions were prepared by dissolving drugs in 100% ethanol, which were then dissolved in sterile saline for intraperitoneal injections, which were given once a day for three months. The above drugs were purchased from MedChemExpress (Monmouth Junction, NJ, USA).

### Cell culture and treatment

The mouse vascular aortic smooth muscle cell line, MOVAS, was purchased from KeyGen Biotech (Nanjing, China). Cells were cultured in high glucose Dulbecco’s Modified Eagle’s Medium (Gibco, Grand Island, NY, USA) supplemented with 10% FBS (CellMax, Beijing, China) and 1% penicillin/streptomycin (Gibco, Grand Island, NY, USA) at 37°C in a humidified 5% CO_2_ atmosphere. Cells were seeded in 6-well plates at a density of 1.0 × 10^4^ cells/cm^2^.

To induce the transformation of MOVAS cells into a macrophage-like phenotype, cells were exposed to estrogen with or without the addition of the following drugs: Tamoxifen, which blocks estrogen action; kenpaullone, a small molecule inhibitor of KLF4; and VH-298, a highly potent inhibitor of the VHL/HIF-α interaction. After the treatment at the required concentration and time, the culture medium was discarded, and cells were collected for further analyses. The above drugs were purchased from MedChemExpress (Monmouth Junction, NJ, USA).

### Measurement of blood pressure and pulse

The blood pressure of the tail artery and the heart rate were measured by the tail-cuff method in a quiet environment after completion of the injections. The tail was preheated to approximately 38°C and fixed in a comfortable position. The compression cuff was placed at the root of the tail, inflated, and pressurized. When the pressure reached 270 mm Hg (1 mm Hg = 0.133 kPa), the pressure reduced automatically and slowly, and the blood pressure and heart rate were recorded by the BP-2000 Blood Pressure Analysis System (Visitech systems, Apex, NC, USA), which was connected to a pressure transducer and an amplifier. The blood pressure and heart rates were measured three times a day for five days for all mice, and the means were calculated and used as the blood pressure and heart rate. The appetite, activity, body temperature, body weight (BW), and other reactions of the animals were observed daily until the end of the experiment.

### Serum analysis

ELISAs were used to analyze mouse serum using the following kits according to the manufacturer’s instructions: E2 (Ruixin Biotech, RXJ203008M), CRP (Ruixin Biotech, RX203183M), IL-6 (Ruixin Biotech, RX203049M), and FCN1 (Meimian, MM-46191M2).

### X-ray angiography

After anesthetization with pentobarbital sodium, the heart was immediately exposed at the left margin of the sternal incision, and the left ventricle was punctured with a 1.0 mL syringe with a needle at the apex of the heart. Then, 0.5 mL of iodoxanol was quickly injected, and the mouse was immediately placed into a small animal X-ray imaging system for vascular imaging. The imaging parameters were 38 kV, 6 s, and 0.2 mA. Vascular stenosis and renal morphology were observed after image collection.

### Histopathological examination

Abdominal aortic tissues were fixed in 10% formalin for 24 h at 4°C and then embedded in paraffin blocks, which were cut into sections (5 μm). The sections were stained with hematoxylin and eosin (HE), Masson’s Trichrome reagent, and Picro-Sirius Red reagent according to standard routine protocols.

### Immunofluorescent staining

Cryosections were fixed for 10 min in ice-cold methanol, washed with phosphate buffered saline (PBS), and blocked with 3% BSA. The sections were then incubated with the following primary antibodies: rabbit anti-ACTA2 (Servicebio, GB111364, 1:500) and mouse anti-CD68 (Santa Cruz Biotechnology, sc-20060, 1:200). After washing, the sections were incubated with the following secondary antibodies: FITC-conjugated goat anti-rabbit (Servicebio, GB22303, 1:300) and Cy3-conjugated donkey anti-mouse (Servicebio, GB21301, 1:300). The sections were then counterstained with DAPI (Boster Biotech, AR1176) to visualize nuclei and imaged using a Leica TCSSP8 DMI8 LASX microscope with Leica LASX software.

### Quantitative real-time PCR

Total RNA of MOVAS cells was extracted using the M5 Universal RNA Mini Kit Tissue/Cell RNA Rapid Extraction Kit (Mei5 Biotechnology Co., Ltd., Beijing, China). The concentration of total RNA was determined with a SpectraMax QuickDrop (Molecular Devices, Shanghai, China) according to the manufacturer’s protocol. cDNA was synthesized using the PrimeScript™ RT Master Mix (Perfect Real Time) (Takara Bio Inc., Beijing, China) and a C1000 Touch Thermal Cycler (Bio-Rad, Hercules, CA, USA) according to manufacturer’s protocols. qRT-PCR was performed using 2X M5 HiPer Realtime PCR Super mix with Low Rox (Mei5 Biotechnology Co., Ltd., Beijing, China) and a QuantStudio 6 Flex Real-Time PCR System (Thermo Fisher Scientific Inc., Waltham, MA, USA). The cycle threshold (Ct) values were normalized to the ACTB gene as the loading control. Relative gene expression levels were calculated using the 2^−ΔΔCt^ method. Each experiment was repeated in triplicate. The primer sequences (Sangon Biotech Co., Ltd., Shanghai, China) used in this study are listed in Table 1.

**Table 1:** Primers designed for quantitative amplification.

**Genes**	**Forward Primer (5′ to 3′)**	**Reverse Primer (5′ to 3′)**
*Actb*	GTGCTATGTTGCTCTAGACTTCG	ATGCCACAGGATTCCATACC
*Esr1*	CCTGGCTGGAGATTCTGATGATTGG	TCCACCATGCCTTCCACACATTTAC
*Vhl*	TGATGGACTTCTGGTTAACCAA	TTTCAGGGTATACACTGGCAAT
*Hif-1α*	GAATGAAGTGCACCCTAACAAG	GAGGAATGGGTTCACAAATCAG
*Klf4*	AGTTTGTGCTGAAGGCGTCTCTG	TGGGCTTCCTTTGCTAACACTGATG
*Cd68*	CCTCTTGCTGCCTCTCATCATTGG	GGCTGGTAGGTTGATTGTCGTCTG
*Lgals3*	TATGACCTGCCCTTGCCTGGAG	CTGTTTGCGTTGGGTTTCACTGTG
*Acta2*	CGTGGCTATTCCTTCGTGACTACTG	CGTCAGGCAGTTCGTAGCTCTTC
*Tagln*	ACTCTAATGGCTTTGGGCAGTTTGG	CCTCTTATGCTCCTGGGCTTTCTTC
*Cnn1*	CAAAGTCAATGTGGGAGTCAAG	CAGTTTGGGATCATAGAGGTGA
*Myh11*	AAGCAGCTAAAGGACAAAACAG	ATGTCACATTGTCATTTAGCGG

### Western blot

Mouse vascular tissue was placed in RIPA lysis solution containing phosphatase and protease inhibitors and thoroughly ground with a tissue crusher. Protein was quantified with a BCA Protein Assay Kit (Boster Biotech, Wuhan, China) according to the manufacturer’s instruction. Proteins were separated by electrophoresis using 10% sodium dodecyl sulfate-polyacrylamide gel electrophoresis (SDS-PAGE) for 1–1.5 h at 130 V. Subsequently, proteins were transferred to polyvinylidene fluoride membranes (Merck Millipore, Burlington, MA, USA) for 1 h at 320 mA. Membranes were blocked with 5% nonfat dry milk/TBST for 1.5 h at room temperature and then incubated with the following primary antibodies overnight at 4°C: GAPDH (Servicebio, GB15004, 1:2000), KLF4 (Santa Cruz Biotechnology, sc-393462, 1:800), ACTA2 (Santa Cruz Biotechnology, sc-53142, 1:800), SM22α (Proteintech, 10493-1-AP, 1:2000), CD68 (Santa Cruz Biotechnology, sc-20060, 1:800), and EMR1 (Proteintech, 27044-1-AP, 1:1000). After washing, the membranes were incubated with the appropriate HRP-conjugated secondary antibody (Boster Biotech, BA1056, 1:8000) for 1 h at room temperature. Protein signals were visualized using PierceTM ECL Western Blotting Substrate (Boster Biotech, Wuhan, China) and a ChemiDoc System (BioRad, Hercules, CA, USA). Densitometric analyses were performed using Image Lab software (BioRad).

### Flow cytometry (FCM) analysis

The expression of the F4/80 macrophage marker in MOVAS cells was detected by FCM. Briefly, MOVAS cells were resuspended in 100 μL of PBS and 2.5 μL of antibody followed by incubation for 0.5 h. Cells were then centrifuged, washed three times with PBS, resuspended in 100 μL of PBS, and analyzed by FCM.

### Transmission electron microscope (TEM) analysis

Tissue samples were prefixed with 3% glutaraldehyde, postfixed in 1% osmium tetroxide, dehydrated in an acetone series, infiltrated in Epox 812, and embedded. Semi-thin sections were stained with methylene blue, and ultra-thin sections were cut with a diamond knife and stained with uranyl acetate and lead citrate. Sections were examined with a JEM-1400-FLASH TEM.

### Statistical analyses

All statistical analyses were performed using GraphPad Prism 8.0 (GraphPad Software, La Jolla, CA, USA), and the results are presented as the mean ± SEM. Testing for normality was performed by the Kolmogorov–Smirnov test with Dallal–Wilkinson–Lilliefors correction. When comparing two groups, nonparametric Mann-Whitney *U*-test or unpaired Student’s *t*-test was used according to the distribution. When comparing three or more groups, ANOVA test was performed with subsequent Dunn’s test and adjustment for multiple comparisons. A two-sided *P* < 0.05 was considered statistically significant.

### Data availability statement

The original contributions presented in the study are included in the article. Further inquiries can be directed to the corresponding authors.

## Supplementary Materials

Supplementary Figures
