# Supplementary material for: High estrogen induces trans-differentiation of vascular smooth muscle cells to a macrophage-like phenotype resulting in aortic inflammation via inhibiting VHL/HIF1a/KLF4 axis
Source: Aging (Albany NY). 2024 Jun 5;16(11):9876–98. doi: 10.18632/aging.205904 (PMC11210252; doi:10.18632/aging.205904)
Supplement: Supplementary Figures [file aging-16-205904-s001.pdf]

SUPPLEMENTARY FIGURES

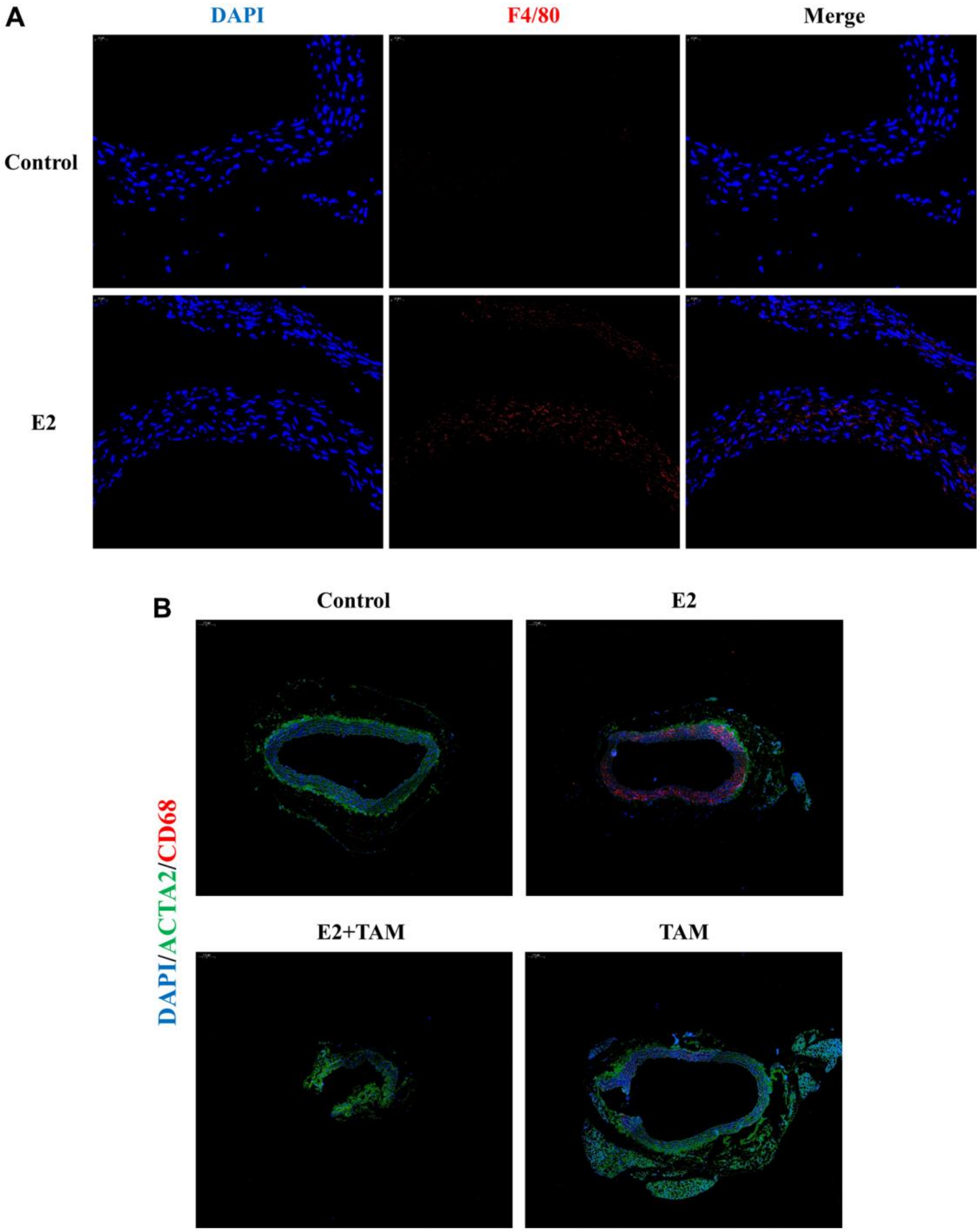

Supplementary Figure 1. Immunofluorescence staining of mouse aortic cell phenotypic markers. (**A**) Mouse aortic tissue sections were done for immunofluorescence staining for macrophage markers (F4/80). Magnification 400×. (**B**) Mouse aortic tissue sections were done for immunofluorescence double staining for smooth muscle cell marker (ACTA2) and macrophage marker (F4/80). Magnification 100×.

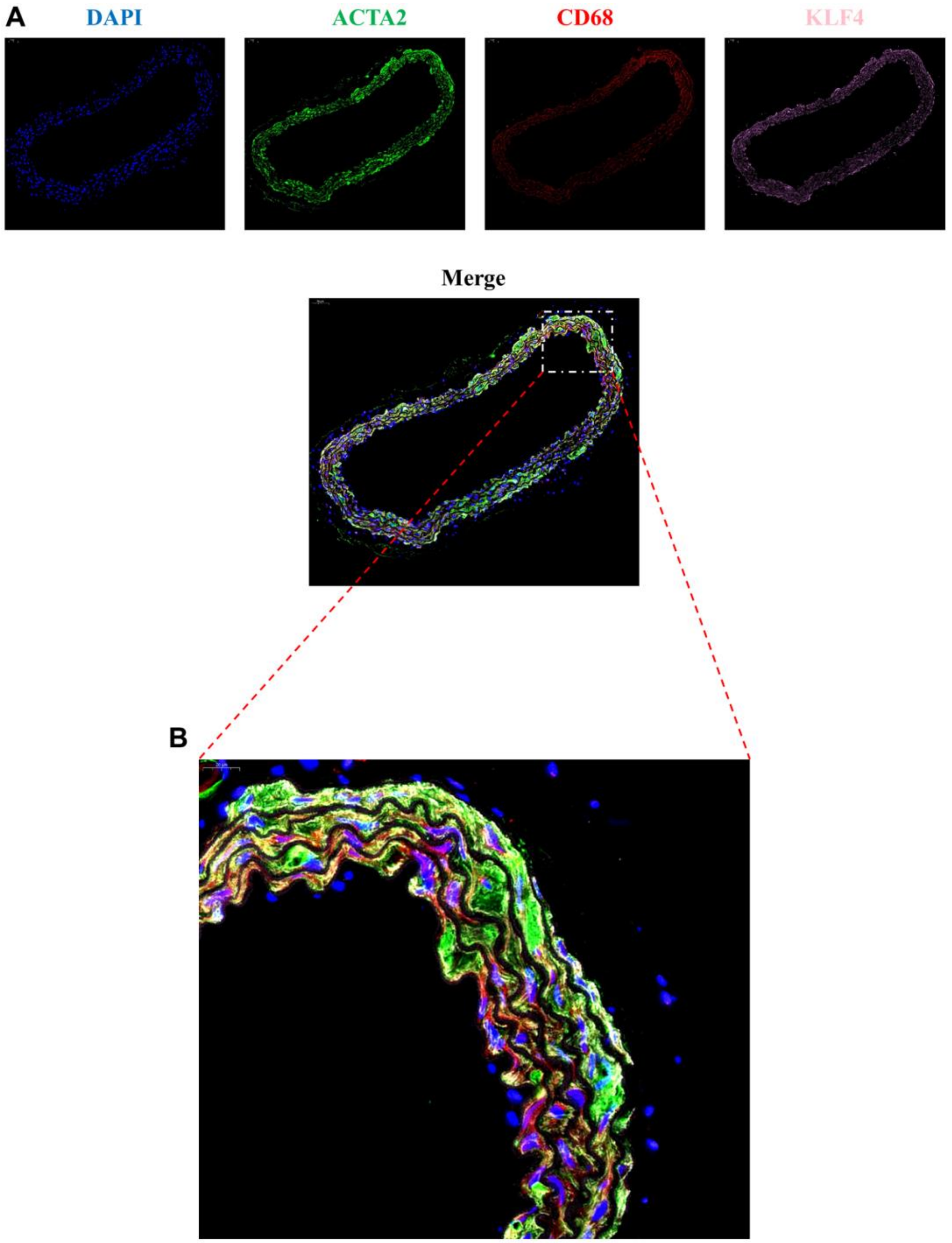

Supplementary Figure 2. Immunofluorescence staining of KLF4, a phenotypic transforming switch in vascular smooth muscle cells. (**A**) Immunofluorescence triple staining for ACTA2, CD68, and KLF4 was done on aortic sections from E2-intervened mice. Magnification 200×. (**B**) Magnification 630×.

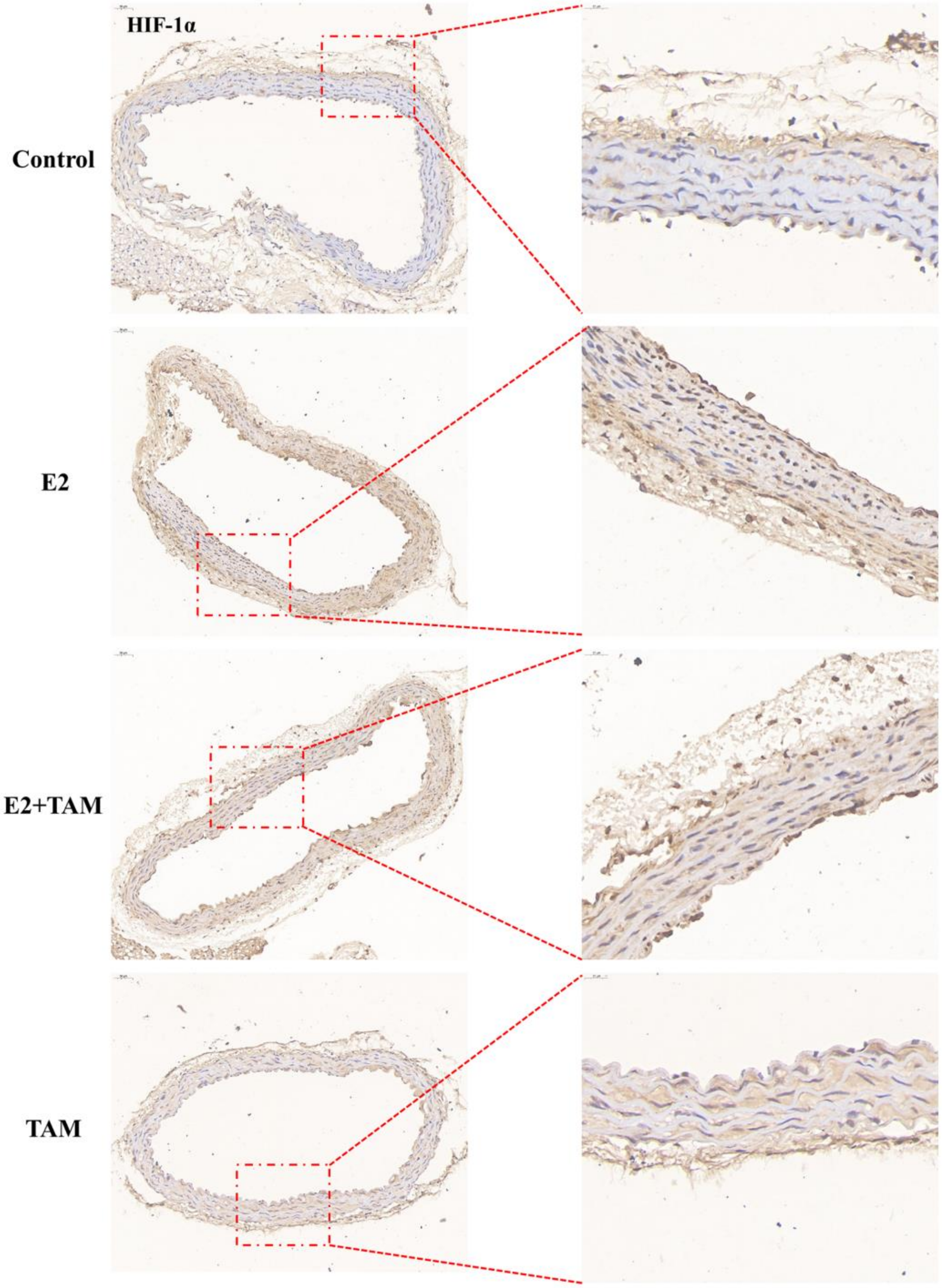

Supplementary Figure 3. Expression of HIF-1α in the aorta of different groups of mice.
